# Supplementary material for: Efficacy of Segmentation for Hyperspectral Target Detection
Source: Sensors (Basel). 2025 Jan 6;25(1):272. doi: 10.3390/s25010272 (PMC11723436; doi:10.3390/s25010272)
Supplement: Supplementary file 1 [file sensors-25-00272-s001.zip › Important annimations.pptx]

## Slide 1
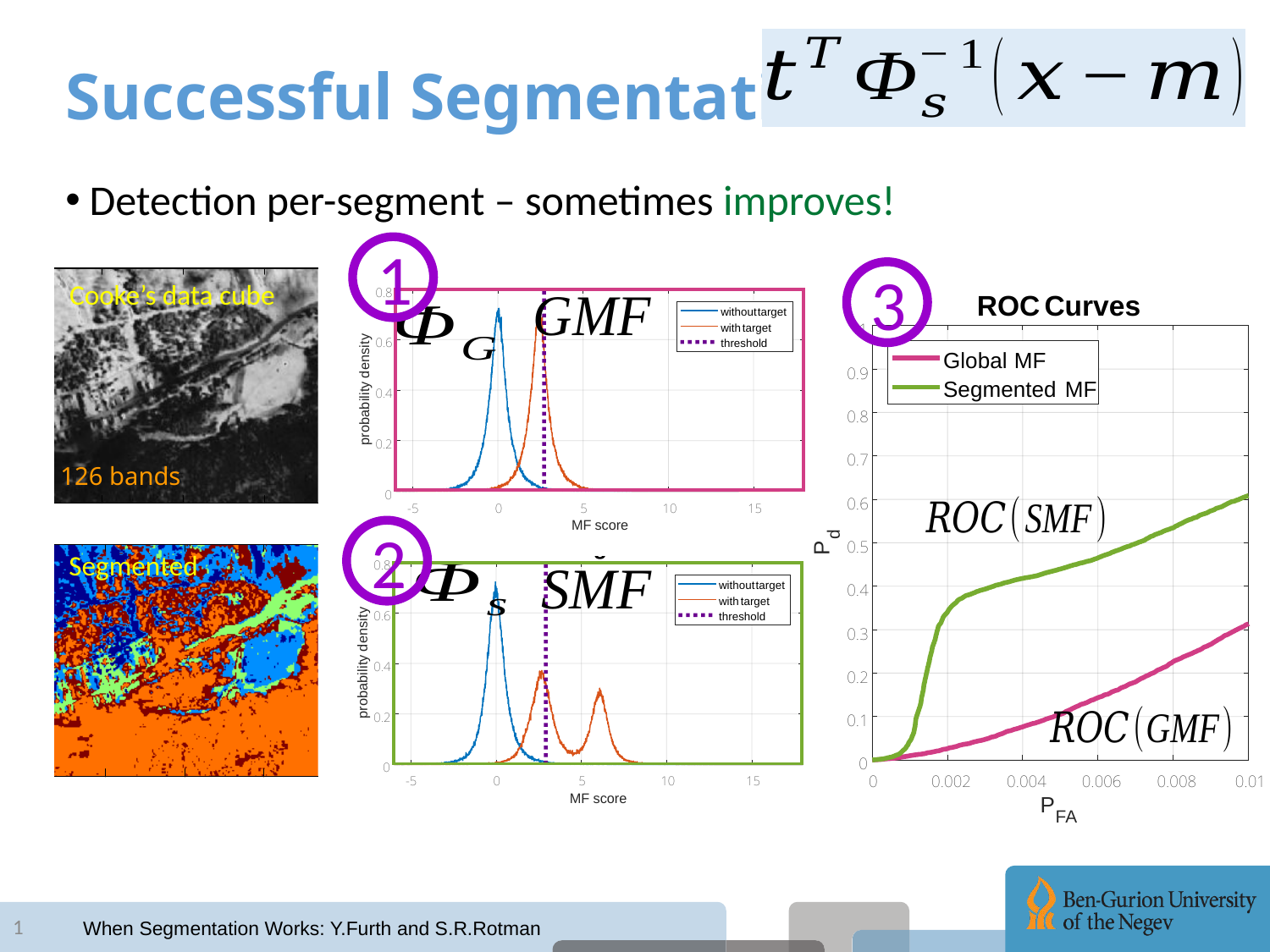

# Successful Segmentation
Detection per-segment – sometimes improves!
1
3
Cooke’s data cube
126 bands
2
Segmented
1

## Slide 2
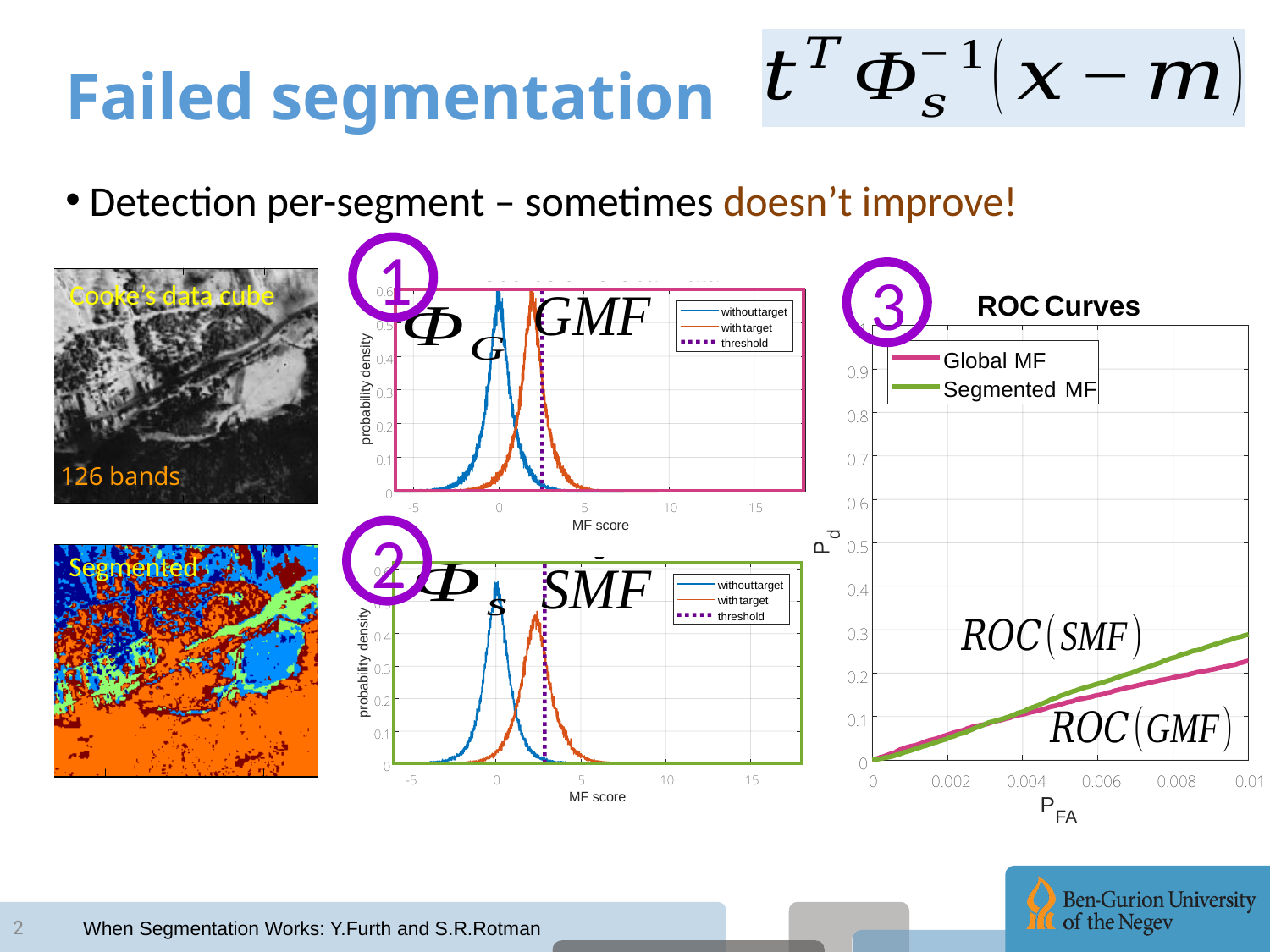

# Failed segmentation
Detection per-segment – sometimes doesn’t improve!
1
3
Cooke’s data cube
126 bands
2
Segmented
2

## Slide 3
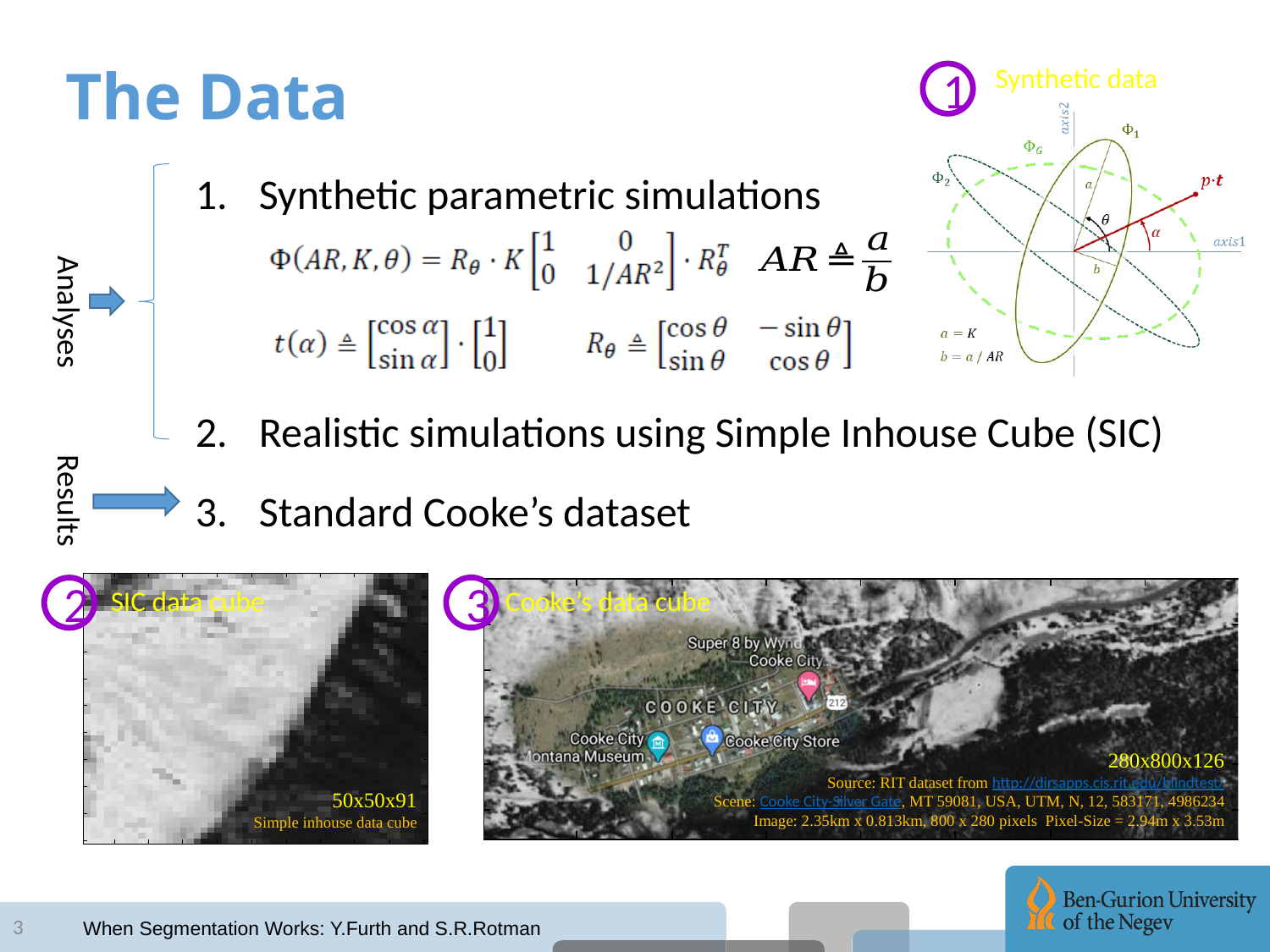

# The Data
Synthetic data
1
Synthetic parametric simulations
Realistic simulations using Simple Inhouse Cube (SIC)
Standard Cooke’s dataset
Analyses
Results
50x50x91
Simple inhouse data cube
SIC data cube
Cooke’s data cube
3
2
3

## Slide 4
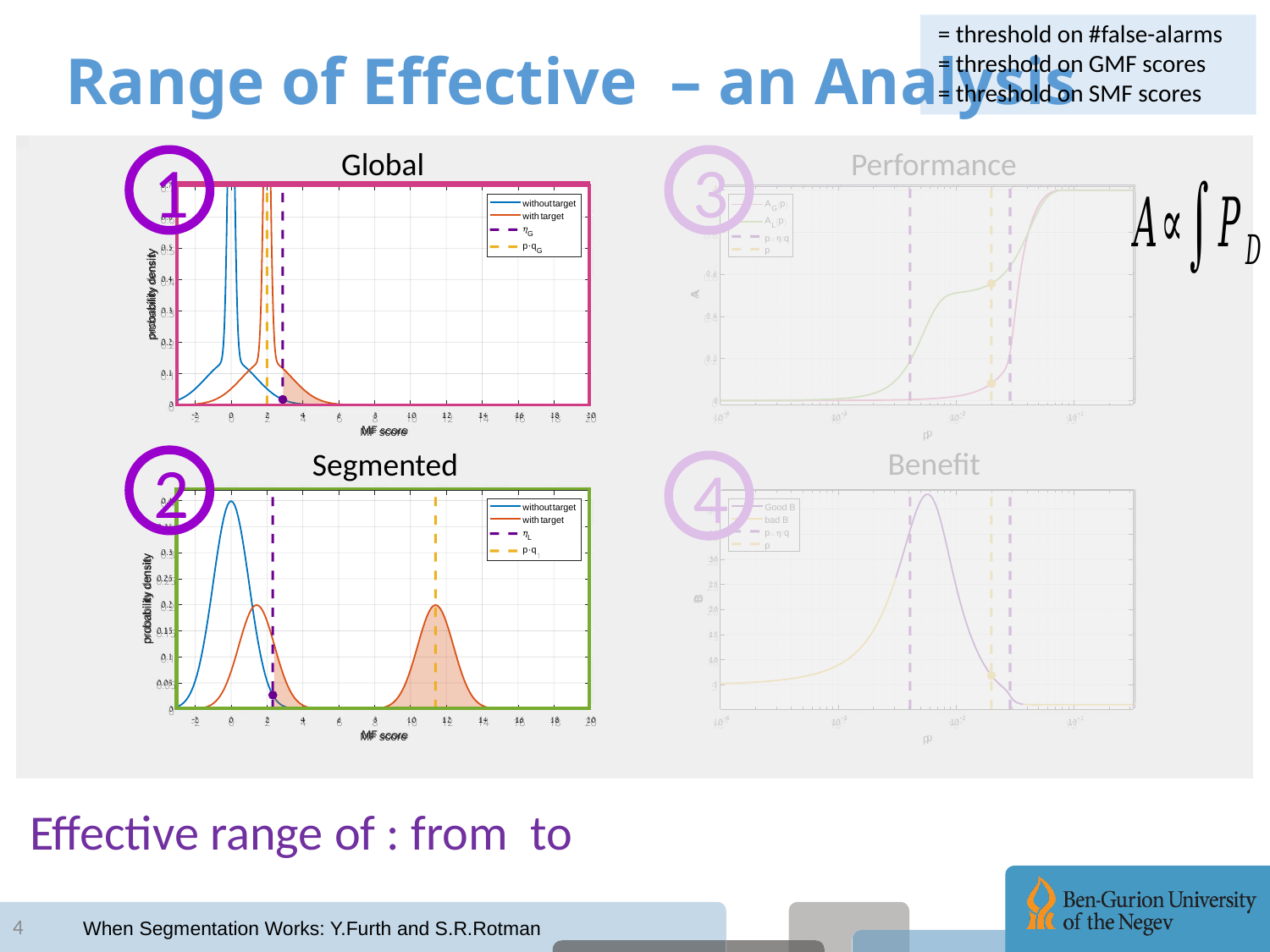

Global
Performance
1
3
Benefit
Segmented
2
4
4

## Slide 5
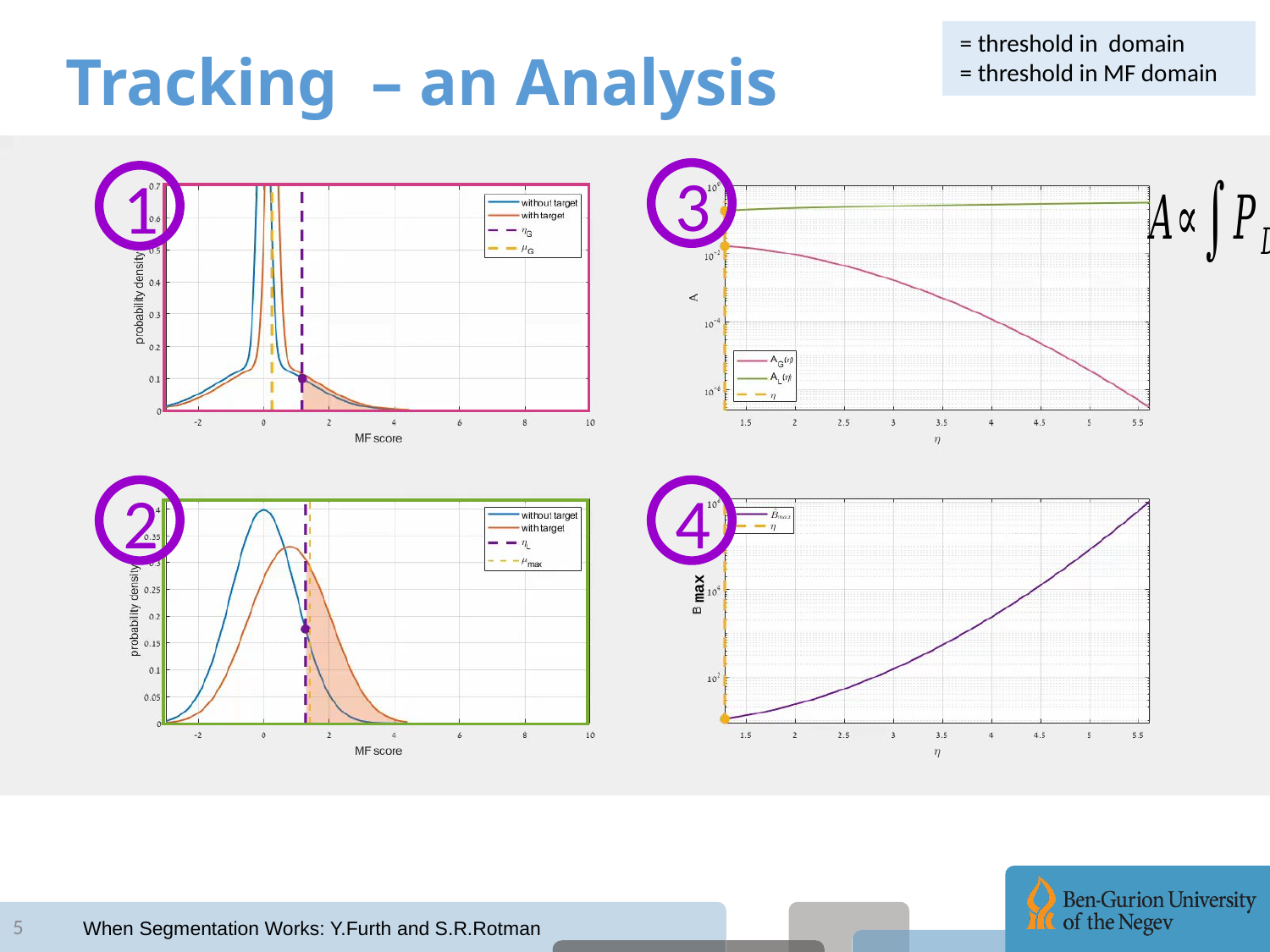

3
1
2
4
max
5

## Slide 6
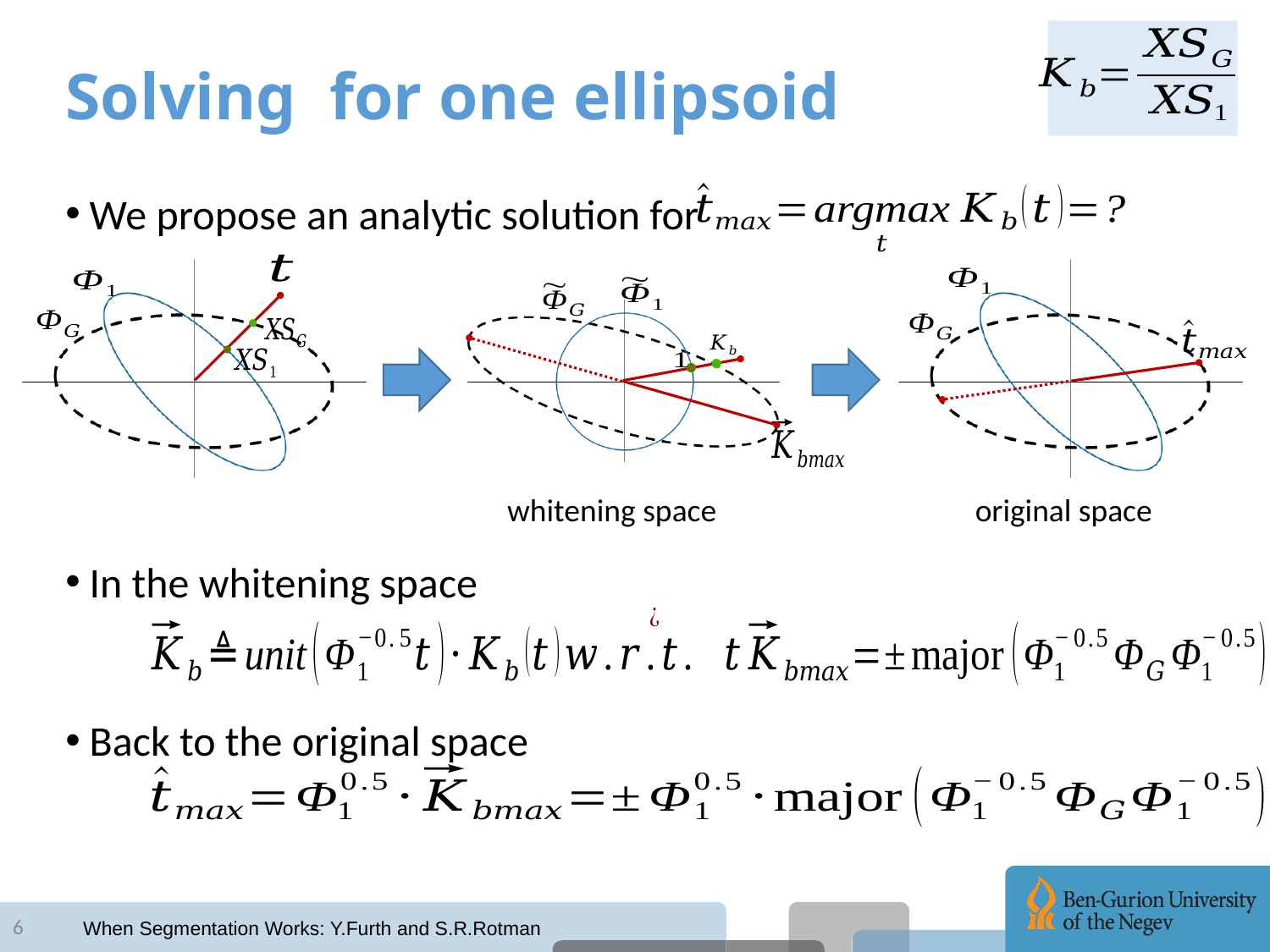

We propose an analytic solution for
whitening space
original space
In the whitening space
Back to the original space
6

## Slide 7
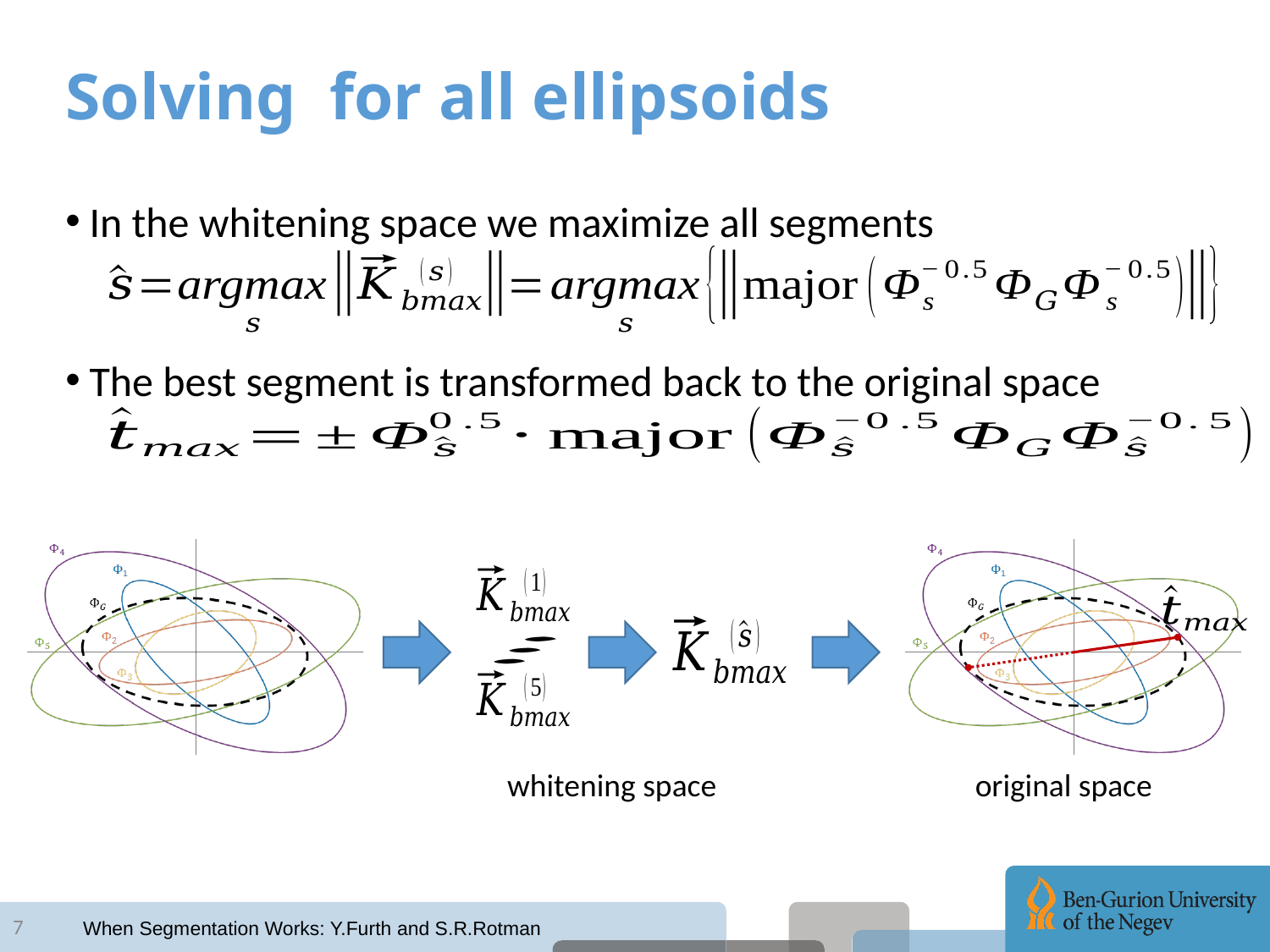

In the whitening space we maximize all segments
The best segment is transformed back to the original space
whitening space
original space
7

## Slide 8
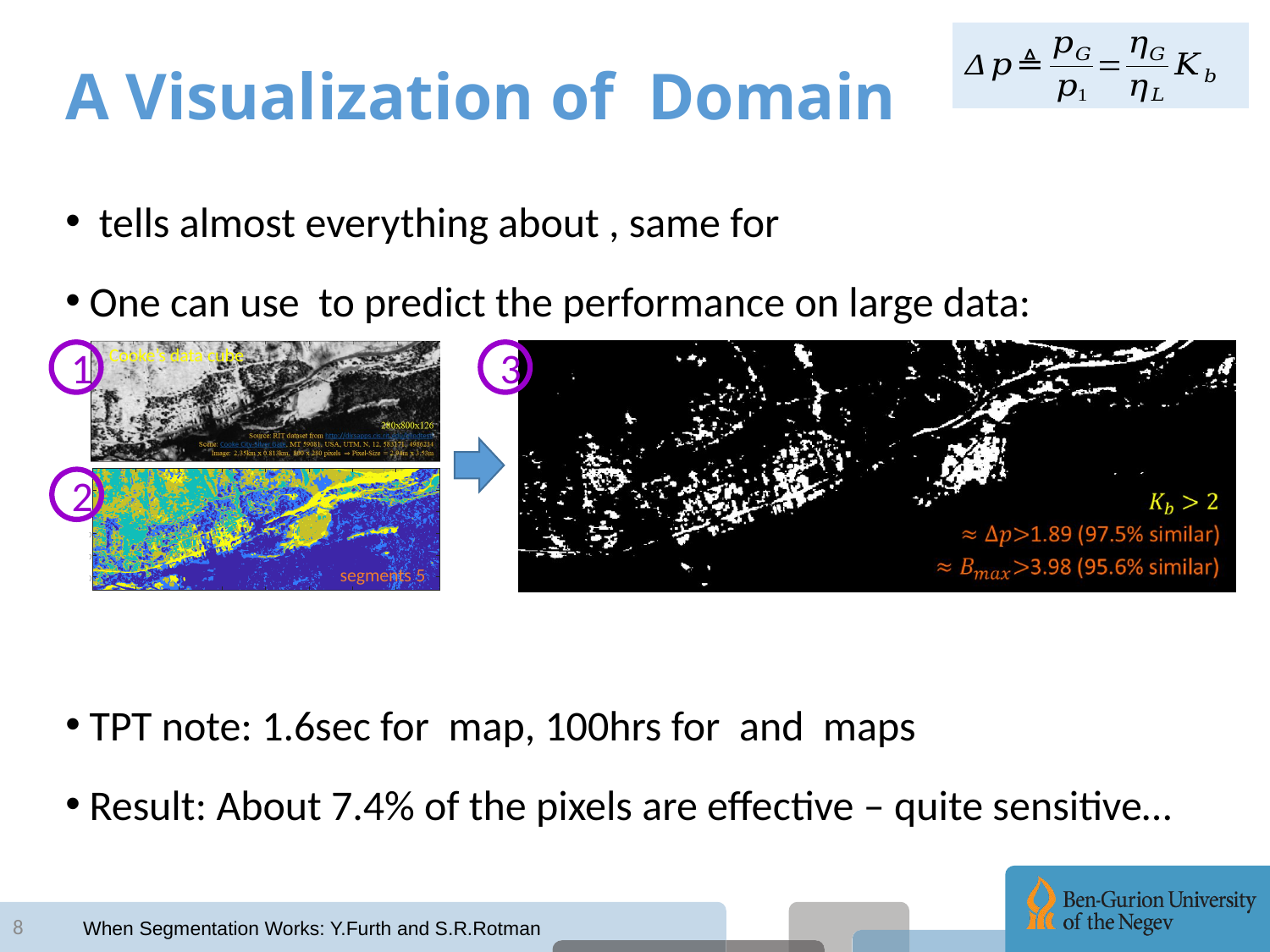

Cooke’s data cube
1
3
2
5 segments
8
